# Supplementary material for: Molecular Cloning and Characterization of MbMYB108, a Malus baccata MYB Transcription Factor Gene, with Functions in Tolerance to Cold and Drought Stress in Transgenic Arabidopsis thaliana
Source: Int J Mol Sci. 2022 Apr 27;23(9):4846. doi: 10.3390/ijms23094846 (PMC9099687; doi:10.3390/ijms23094846)
Supplement: Supplementary file 1 [file ijms-23-04846-s001.zip › ijms-1679272-supplementary.pdf]

Table S1. List of primers used in this study.

Primer Name	Primer Sequence (5'→3')	Purpose
	ATGAATTACATTGCTAACCACGGCG	
MbMYB108-F	TCAAATGCTGCTGAGATGCTGTTGT	full-length cDNA of *MbMYB4*
MbMYB108-R	GCTCTAGAATGGATGCTTTCTCT	full-length cDNA of *MbMYB4*
site-F	CGGGATCCAATGGAAAAACTCC	PCR for restriction site insertion
site-R	GCTAACCACGGCGAAGGCC	PCR for restriction site insertion
MbMYB108-qF	TTCCAGAATCAAAAGCTGTTCTTCA	qPCR
MbMYB108-qR	ACACGGGGAGGTAGTGACAA	qPCR
Actin-F	CCTCCAATGGATCCTCGTTA	qPCR
Actin-R	GGACAGGGTACCCGGGGATCCATGAATTACATTGCTAACCACG	qPCR
HR-F	GCG	PCR for homologous recombination
HR-R	CTGGCATGCCTGCAGGTCGACAATGCTGCTGAGATGCTGTT	PCR for homologous recombination
AtCBF1-F	GTAAG	qPCR
AtCBF1-R	TCGGGACTTTCCAAACCG	qPCR
AtCBF3-F	CCATCTCCTTCGCCGTCAT	qPCR
AtCBF3-R	TCCGGTAAGTGGGTTTGTGAG	qPCR
AtCOR15a-F	AACTCGGCATCTCAAACATCG	qPCR
AtCOR15a-R	CAACAGAGGAATCACCAGCGA	qPCR
AtRD29a-F	CTCTGCTGTCTTGTCGTGGTGT	qPCR
AtRD29a-R	CAACGAGGGGAAGATAAAAGTGT	qPCR
AtNCED3-F	AGCCAGATGATTTTGGAGCCT	qPCR
AtNCED3-R	ATGGCTTCTTCACGGCACGG	qPCR
AtSnRK2.4-F	TTCCTTTGCCCTCGGACG	qPCR
AtSnRK2.4-R	GAGGAAATGGGGATGCAGAT	qPCR
AtCAT1-F	TTCTCACTTCTCCACTTGCG	qPCR
AtCAT1-R	CGCCATGCCGAAAAATACCC	qPCR
AtP5CS-F	CTTGCCTGTCTGAATCCCAGGAC	qPCR
AtP5CS-R	GATACGGATATGGCAAAGCG	qPCR
AtActin-F	CCAAGTCCAAATCGGAAACC	qPCR
AtActin-R	CCCGCTATGTATGTCGC	qPCR
	AAGGTCAAGACGGAGGAT	

```
ATGAATTACATTGCTAACCACGGCGAAGGCCGCTGGAACTCCCTCGCTCGCTGCGCAGGTCTGAAAAGAACTGGAAAGAGCTGCAGATTG
M  N  Y  I  A  N  H  G  E  G  R  W  N  S  L  A  R  C  A  G  L  K  R  T  G  K  S  C  R  L
CGGTGGCTCAACTATCTCCGCCCTGATGTCCGTCGTGGTAACATCAGTCTTGAAGAACAGCTTTTGATTCTGGAACTTCATTCTCGCTGG
R  W  L  N  Y  L  R  P  D  V  R  R  G  N  I  S  L  E  E  Q  L  L  I  L  E  L  H  S  R  W
GGTAACAGATGGTCGAAAATTGCACAGCACTTGCCAGGAAGGACTGACAATGAGATAAAAAACTATTGGAGGACCCGTGTCCAAAAACAT
G  N  R  W  S  K  I  A  Q  H  L  P  G  R  T  D  N  E  I  K  N  Y  W  R  T  R  V  Q  K  H
GCCAAGCAACTCAAATGTGATGTCAACAGCAAGCAGTTCAAGGACACCATGAGATACCTCTGGATGCCTAGATTGGTCGAAAGAATCCAA
A  K  Q  L  K  C  D  V  N  S  K  Q  F  K  D  T  M  R  Y  L  W  M  P  R  L  V  E  R  I  Q
GCCGCCGCGGCCACCTCCACTGCCACTAACGCCACCGCCGCTAATTCCTCTATCGCTGCCCCAGCTACCACTCACCACTTCAACAACAAC
A  A  A  A  T  S  T  A  T  N  A  T  A  A  N  S  S  I  A  A  P  A  T  T  H  H  F  N  N  N
AACATTAACAACTTCCAATCATCAGCACAAATGTTATTACAGCCAAGTCACCATCACTTCGGGGTTTCATCACACTCCCAACTCACACCG
N  I  N  N  F  Q  S  S  A  Q  M  L  L  Q  P  S  H  H  H  F  G  V  S  S  H  S  Q  L  T  P
AGTTACAGTACCCCGGAGAATTCTAGTACGGCGGCCTCATCGGACTCGTTTGGGACTCAGGTATCACCTGTTTCCGACCTCACTGATTAC
S  Y  S  T  P  E  N  S  S  T  A  A  S  S  D  S  F  G  T  Q  V  S  P  V  S  D  L  T  D  Y
TACACCCCTACTACTGCTGCTGCGGCTATCTCGGTTAACAATAACCCTACCCCGGATAATAGTTACTTCGTCCAGGCCAATCATCAAGTT
Y  T  P  T  T  A  A  A  A  I  S  V  N  N  N  P  T  P  D  N  S  Y  F  V  Q  A  N  H  Q  V
GGTACTTACGACAACTACTCGGGGTACTTCGGTAATCAAGGGTTCGGATTAGAACATTTCCAAAACATGGACGAGCAAAACAACAATGAT
G  T  Y  D  N  Y  S  G  Y  F  G  N  Q  G  F  G  L  E  H  F  Q  N  M  D  E  Q  N  N  N  D
CAGTGGGGCACTGTGTACAGTGGGGACATATCGGACAATTGGTGGAATGTTGAGGACATGTGGTTCTTACAACAGCATCTCAGCAGCATTTGA
Q  W  G  T  V  Y  S  G  D  I  S  D  N  W  W  N  V  E  D  M  W  F  L  Q  Q  H  L  S  S  I  *
```

Figure S1. Nucleotide and deduced amino acid sequences of *MbMYB108* gene. The underlined part was that the R2 and R3 conserved domains of *MbMYB108* gene which were also characteristic domains of R2R3-MYB TFs.
